# The Use of the Murphy Button

**Published:** 1895-04

**Authors:** J. B. Murphy

**Affiliations:** Chicago


MURPHY, J. B., CHICAGO: THE USE OF THE MURPHY BUTTON. (*Chicago Clinical Review*).

1. The cicatrix produced with the button does not contract.

2. Size No. ($\frac{3}{4}$ inch, or .02 m.), or No. 2 ($\frac{13}{16}$ inch, or .022 m.) should be used for cholecystenterostomy. I prefer No. 2.

3. End-to-end, side-to-side, and end-to-side of the small intestines should be made with the button No. 3 ($\frac{15}{16}$ inch, or .025 m. in diameter).

4. End-to-end and side-to-side of large intestine should be made with button No. 4 (1 inch, or .026 m. in diameter).

5. A special size ($1\frac{1}{4}$ inch, or .029 m. in diameter), with a long male cylinder, may be used in some cases of resection of the rectum, with advantage, but it should not be used unless it fits loosely.

6. In intestinal obstruction, resection with end-to-end union, gives better results than lateral approximation, and should always be performed where practicable. The same operation should always be done in gangrenous hernia. In fecal fistula, the bowel should be resected and united end-to-end.

7. The patients should receive liquid nourishment as soon as the effect of the anesthetic passes off. The bowels should be made to move as soon as possible after the operation, and frequent evacuations kept up.

8. If the button does not pass in three or four weeks, the rectum should be examined, as it may rest just inside the sphincter.

9. There has been one case reported of occlusion of the button by fecal impaction in the cylinder. This can be easily avoided by a mild cathartic immediately after operation.

10. When returned to the abdomen, the intestines should be placed in parallel lines, especially at seat of approximation, to prevent sharp curves and obstructions. This occurred once with the button; many are reported following suture.

11. There is no danger from obstruction from the button, as not a single case has been reported. This proves that the deductions made by Chaput, of Paris, from experiments on the cadaver, are erroneous.

12. There is no danger of extension of the pressure atrophy beyond the line of pressure.

13. Primary adhesion may be hastened in malignant cases, by abrading the peritoneum with a needle. It is unnecessary in non-malignant cases.

14. A supporting suture is never necessary to secure union, and should only be used to relieve tension when the viscera approximated are forced out of position.

15. The mucous membrane should be pushed down in the cup of the button before closing it; if redundant, it should be trimmed off with the scissors. *It should never be allowed to protrude between the edges of the button, when the button is closed.*

16. While the button is easily inserted, the pathologic conditions requiring the operation may demand the greatest surgical skill to secure a favorable result.

17. The following points, regarding the construction of the button, should be noted before using it:

(1) The spring catches should hold firmly in all positions, and should be made of a metal that will not be corroded by acids.

(2) The elastic pressure cup should be on the male half of the button (never on the female).

(3) The edges of the pressure surfaces should be very smooth and hemispherical in shape.

(4) The spring under the pressure cup should not be too strong.

(5) There have been defective buttons on the market. The following firms are at present manufacturing perfect buttons: J. J. Ryan & Co., Chicago; Truax, Greene & Co., Chicago; Geo. Tiemann & Co., New York; W. F. Ford & Co., New York; Down Bros., London, Eng.; Sharp & Smith, Chicago; Frank, Kratzmueller & Co., Chicago.

18. If the button appears at the opening of the fistula after lateral approximation, do not try to force it through the opening, it is unsurgical; open the abdomen and (*a*) press it back to the nastomotic opening, and through it on down the intestine, and it will pass, or (*b*) make a longitudinal incision in the bowel and take it out.

